# Cell-Surface Proteomics Identifies Lineage-Specific Markers of Embryo-Derived Stem Cells

**DOI:** 10.1016/j.devcel.2012.01.005

**Published:** 2012-04-17

**Authors:** Peter J. Rugg-Gunn, Brian J. Cox, Fredrik Lanner, Parveen Sharma, Vladimir Ignatchenko, Angela C.H. McDonald, Jodi Garner, Anthony O. Gramolini, Janet Rossant, Thomas Kislinger

**Affiliations:** 1 Program in Developmental and Stem Cell Biology, Hospital for Sick Children Research Institute, 555 University Avenue, Toronto, ON M5G 1X8, Canada; 2 Department of Physiology, University of Toronto, 1 King's College Circle, Toronto, ON M5S 1A8, Canada; 3 Department of Molecular Genetics, University of Toronto, 1 King's College Circle, Toronto, ON M5S 1A8, Canada; 4 Ontario Cancer Institute, University Health Network, The Campbell Family Cancer Research Institute, 610 University Avenue, Toronto, ON M5G 2M9, Canada; 5 Institute of Biomaterials and Biomedical Engineering, University of Toronto, 164 College Street, Toronto, ON M5S 3G9, Canada; 6 Division of Cellular and Molecular Biology, Toronto General Research Institute, University of Toronto, 610 University Avenue, Toronto, ON M5G 2M9, Canada; 7 Department of Medical Biophysics, University of Toronto, 610 University Avenue, Toronto, ON M5G 2M9, Canada

## Abstract

The advent of reprogramming and its impact on stem cell biology has renewed interest in lineage restriction in mammalian embryos, the source of embryonic (ES), epiblast (EpiSC), trophoblast (TS), and extraembryonic endoderm (XEN) stem cell lineages. Isolation of specific cell types during stem cell differentiation and reprogramming, and also directly from embryos, is a major technical challenge because few cell-surface proteins are known that can distinguish each cell type. We provide a large-scale proteomic resource of cell-surface proteins for the four embryo-derived stem cell lines. We validated 27 antibodies against lineage-specific cell-surface markers, which enabled investigation of specific cell populations during ES-EpiSC reprogramming and ES-to-XEN differentiation. Identified markers also allowed prospective isolation and characterization of viable lineage progenitors from blastocysts by flow cytometry. These results provide a comprehensive stem cell proteomic resource and enable new approaches to interrogate the mechanisms that regulate cell fate specification.

## Highlights

► Cell-surface proteome for four embryo-derived stem cell lineages ► Comprehensive resource of signaling, adhesion and migration proteins ► Enables isolation of specific cell types during differentiation and reprogramming ► Allows prospective isolation of lineage progenitors directly from blastocysts

## Teaser

Rugg-Gunn et al. provide a large-scale proteomic resource of cell-surface proteins for four embryo-derived stem cell lines: ES, EpiSC, TS, and XEN. Antibodies against lineage-specific cell-surface markers allowed investigation of specific cell populations during differentiation and reprogramming and also prospective isolation of viable lineage progenitors from blastocysts by flow cytometry.

## Introduction

Stem cells derived from early embryos or reprogrammed from somatic cells can be used for the study and treatment of degenerative diseases and hold tremendous promise for the future of regenerative medicine (Murry and Keller, 2008; Yamanaka, 2007). The potential to generate an array of differentiated cell types also raises the opportunity to establish new models of early mammalian development (Rossant, 2008). However, a lack of validated cell-surface markers for flow cytometric analysis and isolation have created road blocks in these fields (Dubois et al., 2011; Van Hoof et al., 2010). For example, major challenges currently faced within regenerative medicine include the assessment of purity of stem cells or stem cell-derived populations, the former to confirm faithful cellular reprogramming and the latter to eliminate the risk posed by introduction of undifferentiated stem cells in vivo. To address this shortcoming, we have examined the cell-surface proteome of the four stem cell lines that are derived from early mouse embryos and applied newly identified protein markers to study differentiation and reprogramming.

The epiblast progenitors (EPIs) of preimplantation blastocysts comprises the pluripotent cells that give rise to all germ layers of the later fetus and are also the tissue source from which embryonic stem (ES) cells are derived (Brook and Gardner, 1997; Evans and Kaufman, 1981; Martin, 1981; Nichols and Smith, 2011). Epiblast stem cells (EpiSCs) are isolated from EPI of early postimplantation embryos and are maintained in a pluripotent state that is distinct from ES cells (Brons et al., 2007; Tesar et al., 2007). The two extraembryonic lineages of the blastocyst also give rise to stable stem cell lines. The outer trophectoderm (TE) layer generates the trophoblast of the placenta and trophoblast stem (TS) cells, and the primitive endoderm (PE) contributes to extraembryonic yolk sac endoderm and gives rise to extraembryonic endoderm stem (XEN) cells (Kunath et al., 2005; Tanaka et al., 1998). Importantly, each stem cell type retains the defining properties and lineage restriction of their in vivo tissue of origin, and therefore provide a useful system in which to study stem cell biology and early mammalian development.

Few cell-surface proteins are known that can distinguish each stem cell type and their in vivo sources within the embryo. Microarray gene expression data from early embryos have been mined successfully to identify two PE-specific cell-surface proteins (Gerbe et al., 2008; Plusa et al., 2008). However, the presence of RNA does not always correlate with the presence of the protein (Cox et al., 2009). Furthermore, a recent study revealed that of all protein classes examined, cell-surface proteins in particular show poor correlation between protein and RNA abundance when comparing cell types (Lundberg et al., 2010). These studies suggest that RNA expression may be an unreliable predictor of cell-specific cell-surface protein expression and that direct proteomic approaches are required to identify protein markers that can distinguish cell types of the early embryo. A large-scale analysis of lineage-specific cell-surface protein expression would also identify those proteins actually involved in important cell signaling, cell adhesion and cell migratory processes during early development and stem cell formation.

We have developed a direct proteomic approach to explore the cell-surface proteome for all four embryo-derived stem cell lines using affinity labeling and mass spectrometry. Antibodies against lineage-specific cell-surface proteins enabled identification and isolation of specific cell populations during stem cell differentiation and reprogramming. Our analysis identified molecules with potential importance in separation and migration of EPI and PE, and of differences in cell signaling between ES cells and EpiSC. Furthermore, cell-surface protein markers allowed prospective isolation and characterization of viable EPI, PE, and TE directly from mouse blastocysts. These results provide a comprehensive stem cell proteomic resource and enable new approaches to interrogate the mechanisms that regulate cell fate specification.

## Results

### Cell-Surface Proteome of Embryo-Derived Stem Cell Lineages

**Figure 1:**
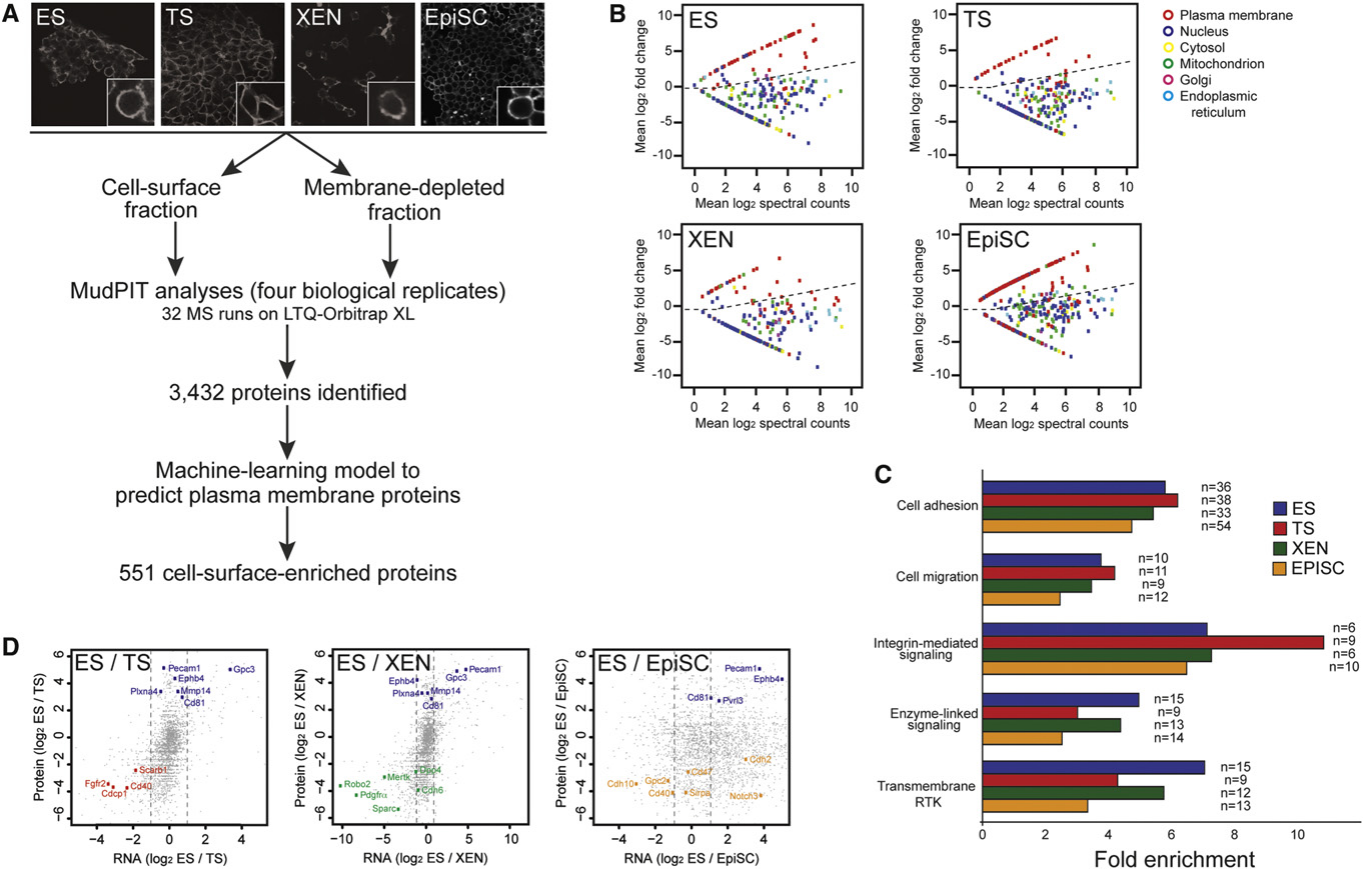
Mapping the Cell-Surface Proteome of Mouse Embryo-Derived Stem Cells (A) Experimental design used to identify cell-surface proteins. Immunofluorescent microscopy reveals that the biotin label is located at the cell surface in all four stem cell lines. See Table S1 for complete protein data set. (B) Scatter plots show mean fold difference in protein abundance based on spectral counting (log_2_ transformed) between the membrane-enriched fraction and whole-cell fraction on the y axis, and the mean protein abundance (log_2_ transformed) from both fractions on the x axis. The proteins displayed are those with GO annotations for cellular location and therefore not all proteins are shown. Dashed lines indicate ratios used to categorize proteins as belonging to the cell surface or not. (C) Functional classification of identified proteins reveals enrichment for biological processes associated with the cell surface. Fold enrichment is relative to whole genome annotations and are highly significant (p < 0.005, Fisher's exact test). See Table S3 for gene ontology data. (D) Scatter plots show difference in protein abundance based on spectral counting (log_2_ transformed; y axis) and RNA expression (log_2_ transformed; x axis) between cell lines for all proteins detected. See Table S4 for individual values. Left panel compares ES and TS cells; middle panel compares ES and XEN cells; right panel compares ES and EpiSC. Cell-surface proteins validated by antibody staining are highlighted. Dashed lines indicate 2-fold change in RNA expression between cell lines.

ES, TS, XEN, and EpiSC were biotinylated with the membrane-impermeable reagent sulfo-NHS-SS-biotin (Figure 1A), which binds to primary amines of cell-surface proteins (Roesli et al., 2006). Individual lysates from biotinylated ES, TS, XEN, and EpiSC were prepared, and biotinylated proteins were affinity captured to collect cell-membrane-enriched protein samples. For comparison, proteins that did not bind to the beads were also collected. These should be depleted in cell-surface proteins but contain cytoplasmic and nuclear proteins (membrane-depleted whole cell samples). Samples were analyzed by mass spectrometry in quadruplicate using a MudPIT approach (Taylor et al., 2009). A total of 3,432 proteins were identified (1,758 for ES; 2,391 for TS; 2,442 for XEN; 2,169 for EpiSC; see Table S1 available online), which represents one of the largest mouse stem cell protein data sets reported (Van Hoof et al., 2006; Wang et al., 2008a).

Gene Ontology (GO) analysis revealed that biotinylated fractions were highly enriched for plasma membrane proteins and depleted in nonplasma membrane proteins (p < 1 × 10^−13^, Fisher's exact test). This suggests that cell-surface proteins had been successfully captured. However, one caveat of chemical labeling strategies and organellar purifications in general is the detection of proteins with annotations other than the organelle of interest (Bergeron et al., 2010), which could hinder the identification of lineage-specific cell surface proteins. To address this challenge, we developed a data mining strategy to predict proteins that are localized to the cell surface. Machine-learning algorithms (Hall et al., 2009b) and training sets of known membrane-localized and non-membrane-localized proteins were used to build a model that categorized each protein as belonging to the cell surface or not (Figure 1B). Applying this stringent model to our data identified 551 proteins predicted to be localized at the cell surface (220 for ES; 222 for TS; 212 for XEN; 416 for EpiSC; Table S2).

As expected, these data revealed a strong enrichment for functional classes that are characteristic of cell-surface proteins, including signaling receptors, cell adhesion and cell migration molecules (Figure 1C and Table S3). The data set contains shared and cell-type-specific proteins within many functional classes, thereby revealing important differences in their protein profiles (Tables 1 and 2). For example, signaling receptors known to be involved in regulating stem cell self-renewal were detected, including Lifr in ES cells, Fgfr2 in TS cells, and Fgfr1 in EpiSC. In addition, Notch receptors were identified in EpiSC, but not in ES cells, suggesting differences may exist between pluripotent cell types. Interestingly, we identified numerous Ephrin and Slit receptors in ES and XEN cells. Their known roles in cell guidance and migration could provide a mechanism to explain the process of cell sorting that occurs during EPI and PE segregation (Brose and Tessier-Lavigne, 2000; Genander and Frisén, 2010; Plusa et al., 2008). Thus, our proteomic data set will provide an important resource of cell-surface proteins that are present on stem cells and could be used in future functional studies to interrogate the mechanisms of self-renewal and differentiation.

### Protein Abundance Is a Reliable Predictor of Cell-Type Specificity

**Figure 2:**
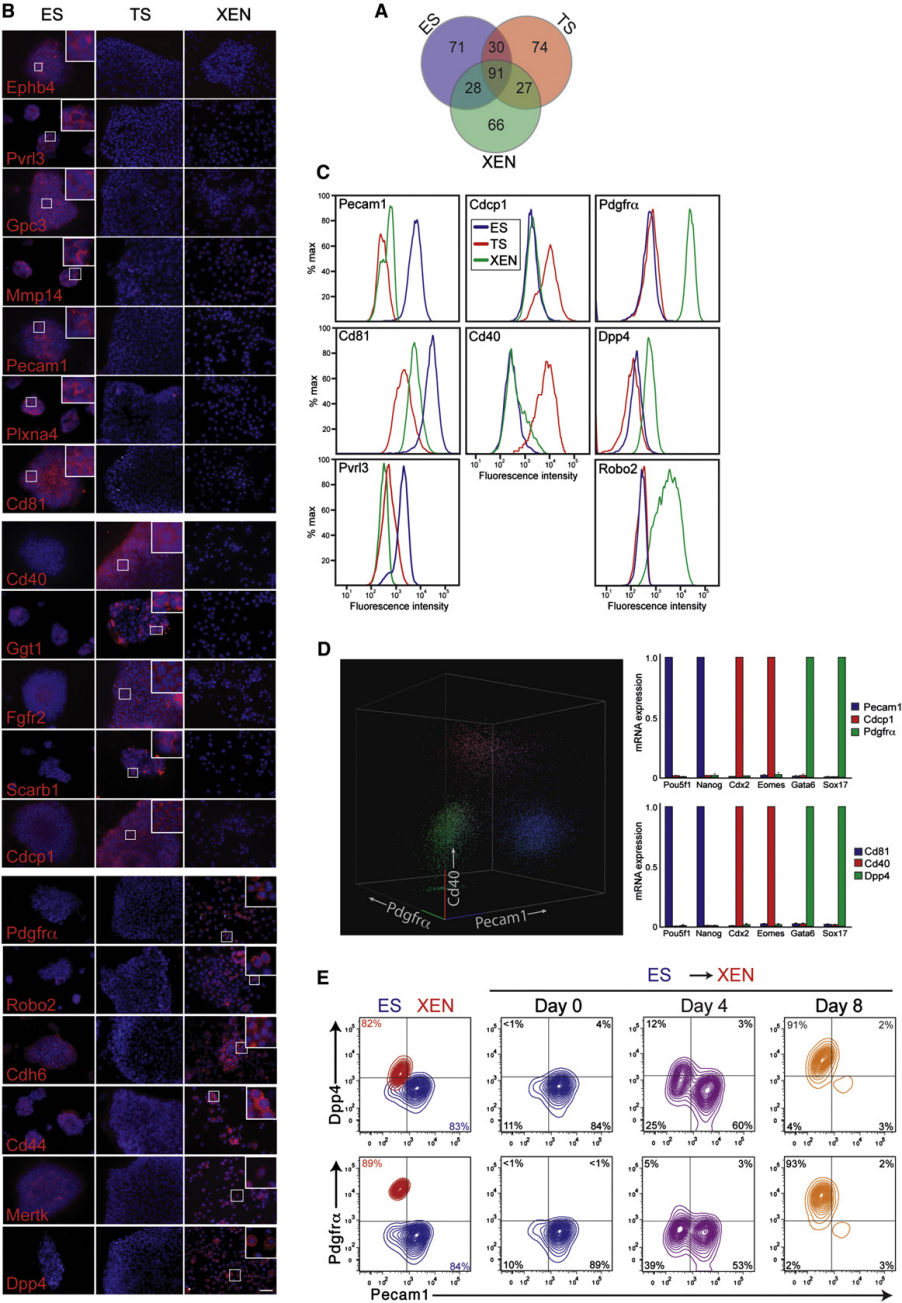
Identified Cell-Surface Proteins Can Be Used to Investigate Cell Fate Changes during Differentiation (A) Venn diagram showing overlap of all cell-surface proteins detected in ES, TS, and XEN cells. See Table S2 for a list of proteins within each category. (B) Immunofluorescent microscopy reveals the cellular localization and cell line specificity of candidate cell-surface proteins. Scale bar, 50 μm. (C) Flow cytometry histograms showing fluorescence intensity of eight cell-surface proteins in individual samples of ES (blue), TS (red) and XEN (green) cells. Data are presented as percentage maximum counts for each sample; 10,000 final counts were recorded for each sample. (D) Representative flow cytometry dot plot showing Pecam1, Cd40 and Pdgfrα expression in a mixed sample containing equal numbers of ES, TS and XEN cells. Cells were sorted by flow cytometry using a combination of antibodies against either Pecam1/Cdcp1/Pdgfrα (upper right) or Cd81/Cd40/Dpp4 (lower right). Sorted cells were analyzed by qRT-PCR for gene expression levels of known lineage-specific transcription factors. Pecam1 and Cd81-positive cells expressed high levels of ES-specific genes *Pou5f1* (also known as *Oct4*) and *Nanog*; Cdcp1- and Cd40-positive cells expressed high levels of TS-specific genes *Cdx2* and *Eomes*; Pdgfrα− and Dpp4-positive cells expressed high levels of XEN-specific genes *Gata6* and *Sox17*. Gene expression levels were normalized to the sample with highest expression. Error bars, SD (n = 3 biological replicates). See Figure S1C for unstained control samples and details of gates used in flow cytometry analysis. (E) ES cell to XEN cell conversion was induced by forcing *Sox17* expression in ES cells. Cell-surface markers of ES (Pecam1) and XEN (Dpp4 and Pdgfrα) were monitored by flow cytometry every four days. Additional cell-surface markers, in addition to validation of XEN cell conversion using qRT-PCR, are shown in Figure S2.

Although previous studies have shown that RNA expression alone can be used to predict cell-specific protein expression (Gerbe et al., 2008; Plusa et al., 2008), the presence of RNA does not always correlate with the presence of the protein (Cox et al., 2009; de Sousa Abreu et al., 2009; Lundberg et al., 2010) suggesting that RNA expression may be an unreliable indicator of cell-specific protein expression. To determine whether the set of cell-specific membrane proteins identified in this current study could have been predicted by RNA expression alone, we integrated genome-wide transcriptional profiles into the protein data set (Table S4). Pairwise comparison of the difference in RNA and protein expression between cell types revealed a subset of cell-surface proteins that would have been identified as cell-specific using transcriptional profiling alone (Figure 1D). However, a larger set of cell-surface proteins showed poor correlation between RNA and protein abundance when comparing cell types and would not be predicted to be cell-specific using transcriptional information alone (Figure 1D). For this set of proteins, RNA transcripts were detected at equivalent (<2-fold difference) levels between cell types, despite robust differences in protein abundance. The cell-specific protein expression of ten proteins within this set was confirmed using antibodies (see below). Disagreement between transcript and protein abundance was prevalent when comparing two cell lines. For instance, discordance between RNA and protein for ES and TS cells occurred for 89/143 (62%) cell-surface proteins. Poor concordance also impacts the reliable identification of cell-specific membrane proteins. Thus, overall only 21 of 178 (12%) cell-specific membrane proteins would have been identified by analysis of RNA expression alone (Table S4). As an example, levels of Pecam1 transcript, a strong marker of ES cells (Robson et al., 2001; Vittet et al., 1996), were similar in ES and TS cells (confirmed by quantitative RT-PCR; data not shown), but Pecam1 protein was detected only in ES cells (34 spectral counts in ES cells, 0 in TS cells; Table S4; confirmed by antibody in Figure 2). Poor concordance between protein and transcript expression for cell-surface proteins are consistent with previous studies and, together with our data, suggest that protein is a more reliable predictor of cell-type specificity than RNA expression alone. These data reinforce the need for a direct proteomic approach for protein marker discovery.

### Cell-Surface Protein Markers Enable Isolation of Lineage-Specific Stem Cells

**Figure 3:**
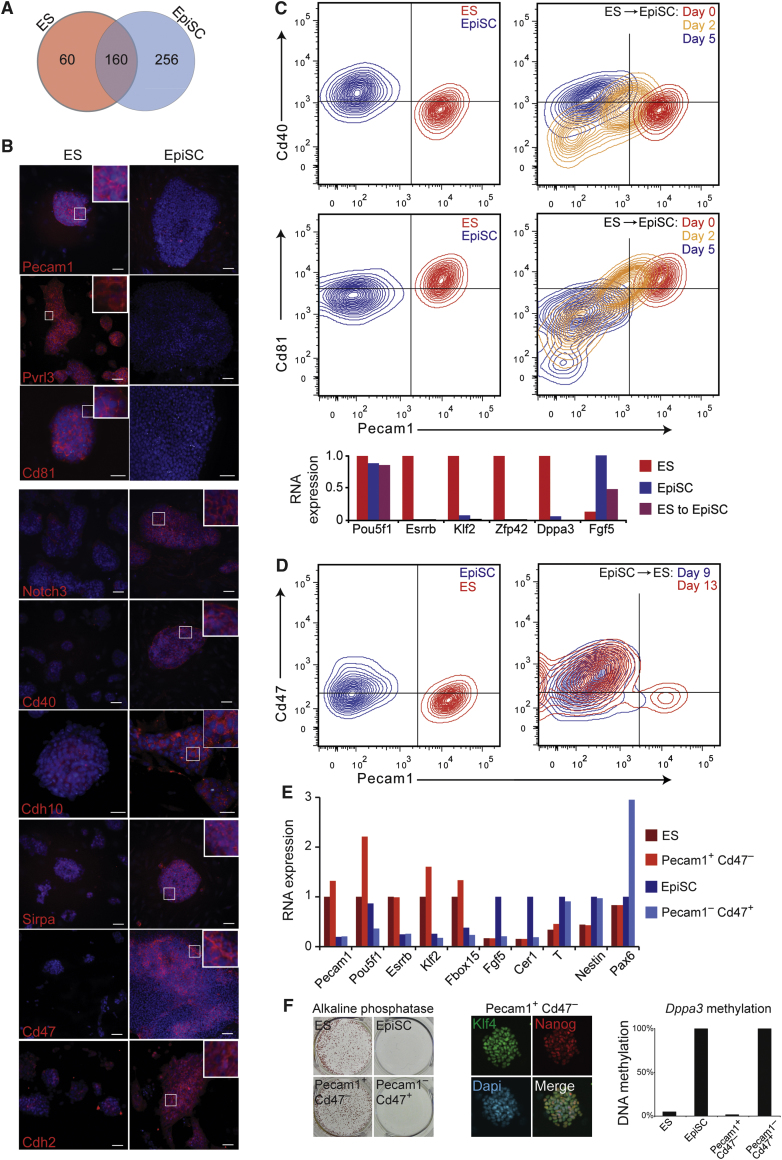
ES cells and EpiSC Can Be Identified and Isolated Using Cell-Surface Markers (A) Venn diagram showing overlap of all cell-surface proteins detected in ES cells and EpiSC. See Table S2 for a list of proteins within each category. (B) Immunofluorescent microscopy reveals the cellular localization and cell line specificity of candidate cell-surface proteins. Scale bar, 50 μm. (C) Flow cytometry contour plots show that ES cells and EpiSC can be distinguished by the cell-surface markers Pecam1, Cd40, and Cd81. The same protein markers allow monitoring of ES cell to EpiSC conversion. Confirmation of cell type by qRT-PCR analysis. (D) EpiSC to ES cell reprogramming was induced by forcing *Nanog* expression in EpiSC and transferring the cells into stringent ES cell conditions (2i/LIF). Changes in cell fate were monitored by flow cytometry using the cell-surface markers Pecam1 and Cd47. Reprogrammed cells (defined as Pecam1 positive and Cd47 negative) were detected on day nine (<0.5% of total population) and on day 13 (1%–5% of total population depending on the experiment). (E) Each cell population was isolated by flow cytometry on day 13 and subjected to qRT-PCR. Reprogrammed cells show similar gene expression profiles to ES cells, but not to EpiSC. (F) Isolated cell populations were transferred separately into media containing 2i/LIF (1,000 cells per well) and resultant colony outgrowths were assayed for markers characteristic of ES cells. Pecam1-positive/Cd47-negative cells displayed alkaline phosphatase activity and Klf4 protein expression, thereby confirming their conversion to an ES cell phenotype. In addition, cells were isolated by flow cytometry on day 12 and the DNA methylation status of the *Dppa3* (*Stella*) promoter was assayed by bisulphite sequencing.

Many cell-surface proteins identified were unique to one or another cell line and provide an important set of lineage-specific markers (Table S2). Comparison between ES, TS and XEN cells revealed 71 cell-surface proteins unique to ES cells, 74 to TS cells and 66 to XEN cells (Figure 2A). Comparison between ES cells and EpiSC revealed 60 cell-surface proteins unique to ES cells and 256 to EpiSC (Figure 3A). We sought to use these protein markers to define a cell-surface protein signature for each cell type that would enable unambiguous detection of specific cells during stem cell differentiation and reprogramming. As an initial step, we screened a panel of commercially available antibodies for those that were able to detect the lineage-specific proteins identified. Of 52 membrane proteins examined, 27 revealed the expected cell-specific cell-surface expression pattern (Figures 2B and 3B; Figure S1) and 25 antibodies failed due to absence of signal in all assays tested or were detected as multiple bands by western blot (see Supplemental Experimental Procedures for antibody details). Of the 27 confirmed proteins, two have been identified previously: Pecam1 in EPI/ES cells and Pdgfrα in PE/XEN cells, and provide further validation of our data (Plusa et al., 2008; Robson et al., 2001; Vittet et al., 1996). To the best of our knowledge, the remaining 25 confirmed cell-surface proteins have not been described previously for ES, TS, XEN, or EpiSC, thereby revealing stem cell-specific expression patterns.

We tested whether the cell-surface proteins, and the antibodies that bind to them, could be used for flow cytometry. Nine antibodies gave strong and cell-specific signals: Pecam1, Cd81 antigen, and Pvrl3 for ES cells; Cdcp1 and Cd40 antigen for TS cells; Pdgfrα, Dpp4, and Robo2 for XEN cells; Cd40 antigen and Cd47 antigen for EpiSC (Figures 2C, 3C, and 3D). Importantly, combinations of these antibodies could separate a mixed population of ES, TS, and XEN cells into individual cell types by flow cytometry (Figure 2D). These results were confirmed using additional ES, TS, and XEN cell lines (data not shown), demonstrating the robustness of these markers. We have, therefore, greatly expanded our knowledge of stem cell specific cell-surface proteins and have identified combinations of antibodies that are able to separate a mixed population of cell types into their individual lineages.

### Analysis of Cellular Reprogramming, ES Cells to XEN Cells

Monitoring the depletion of progenitor cells and the appearance of a new population is critical to optimization of differentiation and reprogramming protocols. To examine this further, we used our cell-specific protein markers to track changes in cell fate during conversion of ES cells into XEN cells. To achieve this, the PE transcription factor *Sox17* was overexpressed in ES, which has been shown previously to promote ES cell to XEN cell conversion (Niakan et al., 2010; Qu et al., 2008; Shimoda et al., 2007). We used a doxycycline-inducible system in order to study early changes in cell state upon *Sox17* expression. Using a panel of six antibodies (Pecam1, Cd81, and Pvrl3 for ES cells; Dpp4, Pdgfrα, and Robo2 for XEN cells) we observed by flow cytometry a complete conversion in cell phenotype within 8–12 days of *Sox17* induction (Figure 2E; Figure S2). The converted cells were indistinguishable by flow cytometry from embryo-derived XEN cells and their change in cell fate was confirmed using gene expression analysis (Figure 2E; Figure S2). Interestingly, on day four, approximately one-third of the cells undergoing conversion were negative for both ES cell and XEN cell markers, indicating that an initial step in the differentiation process is the downregulation of ES cell proteins and this event precedes upregulation of XEN cell proteins. By day eight, the majority (>90%) of cells were positive for XEN cell markers with a minor proportion of negative cells. Together, these data confirm the fidelity of identified cell-specific cell-surface proteins and reveal the temporal and sequential changes in cell state that occur upon transcription factor mediated lineage conversion.

### Cell-Surface Proteins Distinguish ES Cells and EpiSC during Differentiation and Reprogramming

ES cells and EpiSC are pluripotent stem cells that recapitulate the pre- and postimplantation EPI of early mouse embryos, respectively. The two stem cell types differ in terms of gene expression profiles, growth factor requirements, epigenetic status and developmental potency (Rossant, 2008). Better understanding of ES cells and EpiSC is important for identifying how pluripotency is regulated and may also provide clues to explain the differences between human and mouse ES cells, with the former being more akin to EpiSC. Mouse ES cells and EpiSC can be interconverted by alteration of culture conditions augmented by forced expression of key transcription factors such as *Nanog* and *Klf4* (Bao et al., 2009; Greber et al., 2010; Guo and Smith, 2010; Guo et al., 2009; Hall et al., 2009a; Hanna et al., 2010; Silva et al., 2009; Theunissen et al., 2011). However, no cell-surface markers have been shown to functionally isolate the two stem cell types from each other and instead previous reports have relied on transgene expression or cell morphology. Applying our proteomic data set to this deficit, we sought to identify cell-surface proteins that could distinguish between ES cells and EpiSC as this would allow unambiguous identification and quantification during the process of cell conversion.

Our proteomic analysis and subsequent validation by antibodies identified nine cell-specific membrane proteins that are expressed by either ES cells or EpiSC: Pecam1, Pvrl3, and Cd81 antigen for ES cells; Notch3, Cd40 antigen, Cdh10, Sirpa, Cd47 antigen, and Cdh2 for EpiSC (Figures 3A and 3B).

We applied established cell culture conditions to drive the conversion of ES cells into EpiSC (Guo et al., 2009; Zhang et al., 2010). Changes in cell fate during this process were monitored by flow cytometric analysis of Pecam1, Cd81, and Cd40. The flow analysis revealed a progressive change in cell phenotype, whereby ∼75% of cells had downregulated ES cell markers by day two (Figure 3C). By day five, cells were indistinguishable from embryo-derived EpiSC by flow cytometry (Figure 3C). The cells could be maintained in EpiSC culture conditions and revealed a gene expression profile highly similar to EpiSC (Figure 3C), thereby confirming successful cell conversion. These experiments also provided important validation of the identified protein markers and their suitability for analyzing ES cell to EpiSC differentiation.

EpiSC to ES cell reprogramming is an inefficient process (∼1% in published studies) (Guo et al., 2009) and is therefore dependent on the accurate detection and isolation of reprogrammed cells. We transferred *Nanog*-expressing EpiSC into stringent ES cell conditions (termed 2i/LIF) and used flow cytometry to detect the appearance of reprogrammed ES cells. We found that an antibody combination of Pecam1 together with Cd47 or Cd40 provided the most robust readout. Reprogrammed cells (defined here as Pecam1 positive and Cd47 negative) were detected on day nine and this population increased to ∼1%–5% on day 13 (Figure 3D). Each cell population was isolated by flow cytometry and their gene expression profile was analyzed using qRT-PCR. Reprogrammed cells showed expression of ES cell factors *Esrrb*, *Klf2*, and *Fbox15* at similar levels to embryo-derived ES cells and had downregulated EpiSC factors *Fgf5*, *Cer1*, and *T*, suggesting successful reversion (Figure 3E). To test this further, we used flow cytometry to purify reprogrammed cells and transferred the cells into 2i/LIF conditions. The reprogrammed cells formed compact ES cell-like colonies, which were positive for alkaline phosphatase activity and expressed the ES cell factor Klf4, thereby confirming their cellular identity (Figure 3F). In contrast, EpiSC that failed to reprogram (defined here as Pecam1 negative and Cd47 positive) did not upregulate ES cell gene expression profiles or form alkaline phosphatase positive colonies in 2i/LIF (Figures 3E and 3F). Instead, these cells showed upregulation of neural markers *Nestin* and *Pax6* (Figure 3E), which is consistent with a previous study that showed neural induction after EpiSC treatment with FGF inhibitors (Greber et al., 2010). Lastly, we examined whether the cells had undergone epigenetic reprogramming by examining the methylation status of the *Dppa3* (also known as *Stella*) promoter region, which is highly methylated in EpiSC and unmethylated in ES cells (Hayashi et al., 2008). Bisulphite sequencing revealed that reprogrammed cells isolated by flow cytometry on day 12 had an unmethylated *Dppa3* promoter, whereas EpiSC that failed to reprogram remained fully methylated (Figure 3F). The reprogrammed cells, therefore, share molecular features with ES cells and not with EpiSC. Taken together, these studies have identified a panel of cell-surface protein markers that are able to distinguish ES cells and EpiSC during differentiation and reprogramming. These results now enable the accurate detection and isolation of specific cell populations without the need to use transgenic reporter cell lines.

### Identified Cell-Surface Proteins Are Expressed in Lineage-Appropriate Manner In Vivo

Better understanding of the molecular determinants of cell fate decisions and the precise timing of lineage restriction during early embryo development is essential for effective use of stem cells. Progress toward understanding these issues is contingent on the ability to prospectively isolate and characterize each cell lineage directly from blastocysts; however this remains a major technical challenge. Our panel of validated ES, TS, and XEN cell-surface proteins and antibodies present an opportunity to establish conditions that could enable these new approaches.

**Figure 4:**
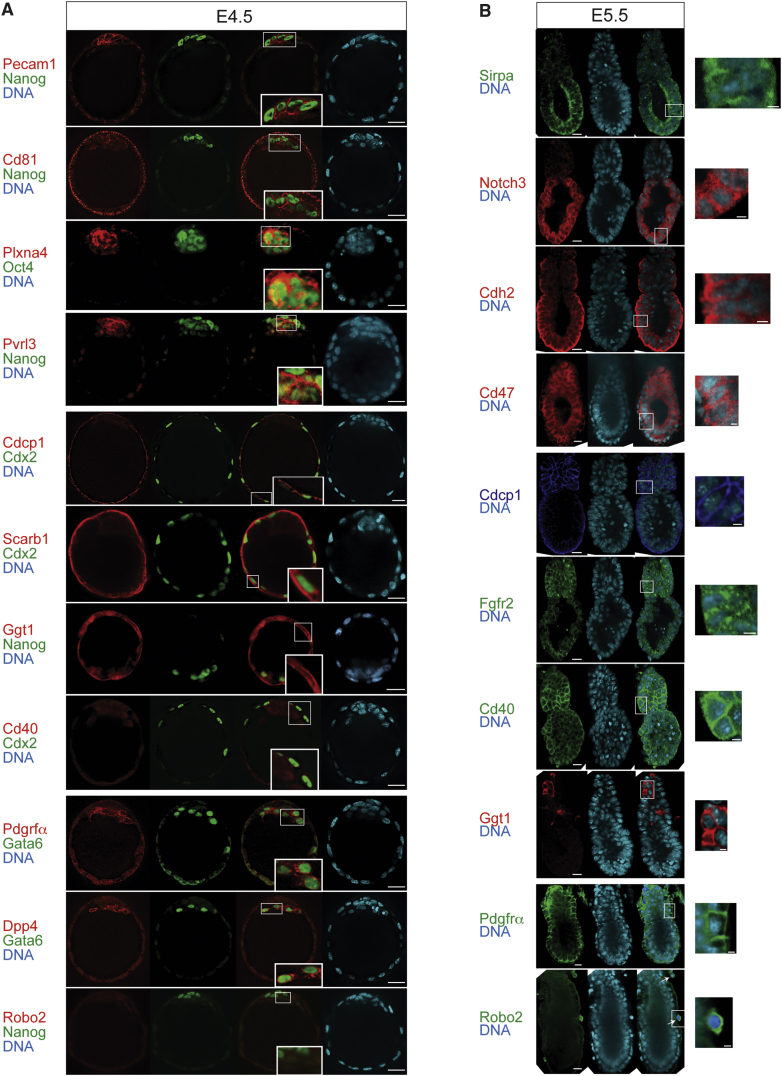
Immunofluorescent Confocal Microscopy of Candidate Cell-Surface Proteins in Mouse Embryos (A) At E4.5, Pecam1, Cd81, Plxna4, and Pvrl3 expression was restricted to Nanog/Oct4-positive EPI cells; Cdcp1, Scarb1, and Ggt1 to Cdx2-positive TE cells; and Pdgfrα and Dpp4 to Gata6-positive cells. Robo2 and Cd40 was not detected at this developmental stage. Scale bar, 20 μm. (B) At E5.5, Sirpa, Notch3, Cdh2, and Cd47 were expressed in EPI; Cdcp1, Fgfr2, Cd40, and Ggt1 in trophoblast; and Pdgfrα and Robo2 to visceral endoderm and parietal endoderm (arrows), respectively. Scale bar, 20 μm. Boxed sections indicate the enlarged regions (scale bar, 5 μm.). The visceral endoderm was removed from some E5.5 embryos to improve antibody accessibility to EPI and trophoblast tissues.

We investigated whether the identified cell-surface proteins were expressed by their in vivo tissue of origin. Embryos were examined by immunofluorescence and costained with known lineage markers Nanog, Oct4, Cdx2, and Gata6 to verify the identity of each cell type (Figure 4A). In embryonic day E4.5 blastocysts, ES cell markers Pecam1, Cd81, Plxna4, and Pvrl3 localized to the cell surface of EPI with no signal detected in PE or TE (Figure 4A). XEN cell proteins Pdgfrα and Dpp4 were restricted to PE with no expression detected in EPI or TE, and TS cell proteins Cdcp1, Ggt1, and Scarb1 localized specifically to TE (Figure 4A). Curiously, Robo2 and Cd40 were not detected at this stage of development (Figure 4A). We therefore examined E5.5 embryos and found that the XEN cell protein Robo2 was expressed by parietal endoderm cells, which are a specific cell type derived from PE (Figure 4B). In addition, the TS cell protein Cd40 was detected throughout the trophoblast of E5.5 embryos, thereby revealing a strong stage-specific expression pattern (Figure 4B). Cd40 is also a protein marker of EpiSC, and consistent with this, we detected cell-surface expression of Cd40 in EPI at E5.5 (Figure 4B). Further examination of additional EpiSC protein markers revealed Sirpa, Notch3, Cdh2, and Cd47 localized specifically to EPI at E5.5 (Figure 4B). In contrast, ES cell proteins Pecam1, Plxna4, and Pvrl3 were not detected in E5.5 embryos (data not shown), confirming that the stage-specific fidelity of protein markers that are able distinguish between ES cells and EpiSC in vitro are also maintained in vivo. Overall, the results validate our approach of using cell lines as models for identifying proteins in cell-types that are not directly amenable to proteomic studies, such as tissues in the early embryo. Sixteen proteins that we identified using the stem cell lines were expressed by the expected embryo lineage, thus revealing previously unappreciated expression patterns in the embryo and also providing potential cell-surface markers for prospective cell isolation.

### Prospective Isolation of Lineage-Specific Cells Directly from Blastocysts by Flow Cytometry

**Figure 5:**
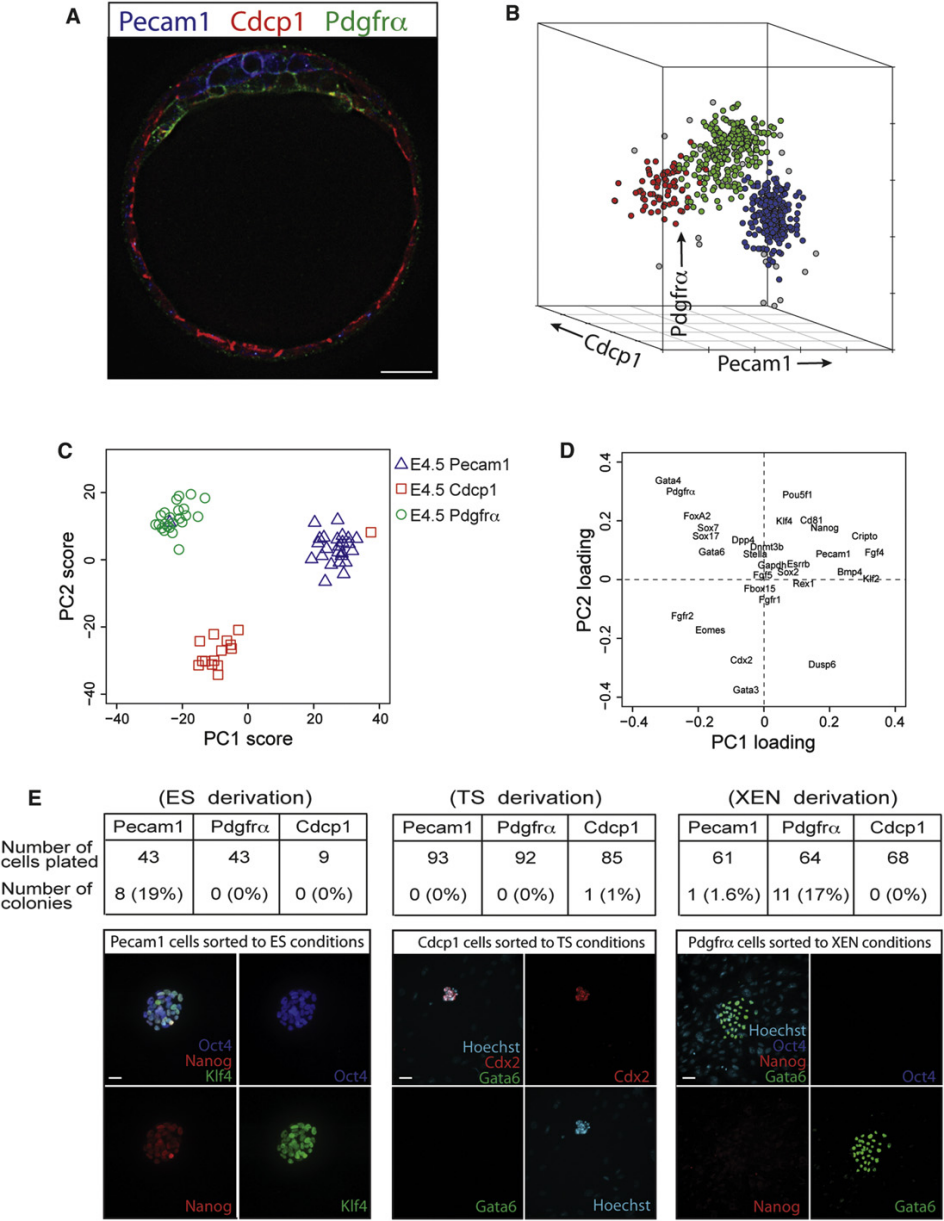
Prospective Isolation of Cell Lineages from Mouse Blastocysts by Flow Cytometry (A) Immunofluorescent confocal section of Pecam1 (blue), Cdcp1 (red), and Pdgfrα (green) in E4.5 blastocysts. Scale bar, 20 μm. (B) Flow cytometry dot plot showing Pecam1, Cdcp1, and Pdgfrα expression in cells isolated from E4.5 blastocysts. The combined data from three independent experiments are shown. Computational analysis of the flow cytometry data defined three distinct cell populations, colored blue, green and red; gray dots represent statistical outliers. (C) Principle component (PC) projections of single cell gene expression profiles. Cells (n = 63) were isolated from E4.5 blastocysts using antibodies to Pecam1, Pdgfrα, and Cdcp1. The first PC is able to discriminate between EPI and TE/PE, the second PC between TE and PE/EPI. (D) PC projections showing the contribution of each gene to the first two PCs. *Fgf4*, *Klf2*, *Cripto*, and *Nanog* are enriched in Pecam1-positive EPI, *Gata4*, *Sox17*, *FoxA2*, and *Gata6* are enriched in Pdgfrα-positive PE, and Gata3, *Cdx2, Eomes*, and *Fgfr2* are enriched in Cdcp1-positive TE. See Figure S3B for heat-maps of expression data. (E) E4.5 blastocysts were sorted by flow cytometry using a combination of Pecam1/Cdcp1/Pdgfrα antibodies and each cell population was separately plated into either ES, XEN, or TS cell derivation conditions. The resulting cell colonies were categorized as ES, TS, or XEN cells based upon expression of lineage markers. Scale bar, 50 μm.

We next examined whether it is possible to sort E4.5 blastocysts into separate lineages using the protein/antibody combinations identified (Figure 5A). To achieve this, there were several significant technical hurdles to overcome. We first determined conditions that could dissociate blastocysts into single cells while maintaining cell viability (see Experimental Procedures). Batches of 30–50 embryos were processed per experiment and ∼25% of cells were recovered after single cell dissociation (∼5–15 cells from each blastocyst). Cells were labeled with antibodies and subjected to flow cytometry. Cell viability was ∼70% (based on propidium iodide staining) and ∼30% of cells were recovered after flow cytometry. An unbiased computational analysis of the flow cytometry data defined three distinct cell populations from blastocysts, based upon the fluorescent intensity of each antibody (Figure 5B). We noticed that the proportion of TE cells was reduced from ∼75% in the blastocyst to ∼15% of cells after flow cytometry, with the remaining cells comprising equal proportions of EPI and PE (Figure S3A). The reduction in TE number was due to the difficulty in obtaining single viable cells, however despite the lower numbers, sufficient TE cells were obtained for analysis in each flow cytometry experiment. These data indicate that each cell lineage had been successfully isolated, thereby representing a significant advance in our ability to analyze specific cell types within the early embryo.

To confirm the lineage identity of each cell population, we sorted E4.5 blastocysts by flow cytometry and subjected individual cells within each population to quantitative gene expression analysis using the BioMark Fluidigm System. Principle component analysis revealed that Pecam1-positive (n = 25), Pdgfrα-positive (n = 23), and Cdcp1-positive (n = 15) cells formed three distinct clusters and each cell type could be unambiguously identified based upon expression levels of known EPI, PE, and TE genes (Figures 5C and 5D; Figure S3B). The clear separation of lineage-specific transcription factor expression suggests that each cell lineage is fully segregated in blastocysts at E4.5. These data also provide an estimate of the error rate during cell sorting. One EPI cell was falsely allocated into the PE population, and one TE cell that was falsely sorted into the EPI population, resulting in an error rate of ∼4%. An alternative combination of antibodies, including Cd81 and Dpp4, showed a similar trend in lineage-specific gene expression levels but the cell populations were less distinct (Figure S3C).

Lastly, we assessed whether cells isolated from E4.5 blastocysts by flow cytometry were viable, as this would enable sorted cells to undergo functional assays. To test this, each sorted cell population was transferred separately into ES, TS, and XEN cell derivation conditions. Importantly, cells isolated from all three lineages remained viable after 96 hr in culture. Differences in stem cell derivation efficiency between each isolated population also provide insight into the lineage restriction of each blastocyst cell type. ES cell colonies emerged from the Pecam1 population (EPI cells; efficiency of 19%) but no ES cell colonies developed from Cdcp1 (TE cells) or Pdgfrα (PE cells) populations (Figure 5E). Conversely, numerous XEN cell colonies emerged from the Pdgfrα population (efficiency of 17%), but only one XEN cell colony from Pecam1 cells (efficiency of 1.6%) and none from Cdcp1 cells (Figure 5E). From Cdcp1-positive cells, we obtained one TS cell colony (efficiency of 1%), whereas no TS cells emerged from Pecam1 or Pdgfrα-positive cells (Figure 5E). The low derivation efficiency of TS colonies from Cdcp1-positive cells is not unexpected, as even established TS cell lines have lower clonal efficiency than ES and XEN cells (data not shown). These data confirm that cells remain viable after embryo dissociation and flow cytometry, thereby enabling functional studies to be applied. Furthermore, each cell type only gave rise to the appropriate stem cell lineage, indicating that EPI, PE, and TE are lineage restricted in E4.5 embryos even when transferred into conditions that are strongly selective for alternate stem cell lineages.

## Discussion

We have developed a proteomic and bioinformatic strategy to discover cell-surface proteins that are present on embryo-derived stem cells, using an established cell-surface labeling strategy (Borgia et al., 2010; Faça et al., 2008; Schliemann et al., 2010). We then screened a large panel of antibodies and found 27 cell-surface proteins with lineage specificity. Each of the four stem cell lines derived from the early mouse embryo now has a defined cell-surface protein signature. We applied this set of proteins/antibodies to several critical problems currently encountered during in vitro differentiation and reprogramming. Examination of ES cell to XEN cell differentiation, and ES cell to EpiSC interconversion, confirmed the utility and specificity of our protein markers and extended our understanding of these cellular processes. In regards to ES cells and EpiSC, previous studies relied on transgene expression or the judgment of cell morphology to detect reprogrammed cells during EpiSC to ES cell conversion (Bao et al., 2009; Greber et al., 2010; Guo and Smith, 2010; Guo et al., 2009; Hall et al., 2009a; Hanna et al., 2010; Silva et al., 2009; Theunissen et al., 2011; Zhang et al., 2010). Here, we present cell-surface markers that can distinguish these two closely related pluripotent cell types. It is likely that the identified protein markers could also be used to study other reprogramming events, such as the conversion of somatic cells to induced pluripotent stem cells, which closely resemble ES cells (Yamanaka, 2007).

Identifying mechanisms that regulate lineage restriction in the embryo is essential for understanding cell fate decisions during development and for improved control over stem cell differentiation. Characterization of early embryo cells is also important for providing a baseline to which cells can be compared after reprogramming in order to better define their cellular phenotype (Rossant, 2008). In keeping with these concepts, we show that the majority of the cell line-specific proteins are expressed in a lineage-appropriate manner in early mouse embryos. Our proteomic resource also contains information on the differences in expression of important cell-surface ligands and receptors that can be mined to understand lineage development, differentiation and reprogramming. In particular, interactions between cell-surface proteins and extracellular ligands are key to regulating cell behavior. Among the candidate proteins detected, the expression of multiple Ephrin receptors in ES cells (Epha2/4, Ephb2/3/4) and the Slit receptor Robo2 in the XEN cells is particularly intriguing, considering their known roles in guidance, migration and control of stem cell proliferation (Brose and Tessier-Lavigne, 2000; Genander and Frisén, 2010). This could suggest a potential role for Ephrin and Slit pathways during the process of cell sorting that occurs during EPI and PE segregation in the blastocyst (Plusa et al., 2008). In addition, identification of CUB domain-containing protein 1 (Cdcp1) as a trophoblast-specific cell-surface protein raises the possibility that Cdcp1 may be converting extracellular information into intracellular signaling pathways, which is a key role for Cdcp1 in other epithelial cell types (Wortmann et al., 2009). Thus, our proteomic resource will provide a valuable set of protein targets for future studies.

Gene expression profiles of EPI, TE, and PE have been obtained by retrospective analyses of individual cells after blastocyst disaggregation (Guo et al., 2010; Kurimoto et al., 2006). These studies revealed that cells from each lineage in the blastocyst could be distinguished according to their expression profiles. Our demonstration of prospective sorting of EPI, TE, and PE cells has several major advantages, including a priori classification of cell types, grouping of cells for population studies and the recovery of viable cells for functional characterization. This approach enabled us to quantify gene expression levels of key transcription factors during lineage specification and to perform functional in vitro assays to demonstrate that cells expressing EPI, PE, or TE markers in blastocysts at E4.5 were lineage restricted. Our results are consistent with current models of mouse development (Bruce and Zernicka-Goetz, 2010; Lanner and Rossant, 2010) and reveal that full segregation of EPI, PE, and TE cells has occurred by E4.5. This methodology enables direct access to the individual cell lineages of the early embryo and isolation of viable lineage progenitors. Further analysis of single cells by RNA sequencing and chimera formation should accelerate investigation of early mammalian development.

The proteomic strategy described here should be broadly applicable to other developmental and stem cell systems, especially when combined with current large-scale efforts to generate antibody libraries (Uhlén et al., 2005). Future work to enhance our proteomic resource could include alternative methodologies, including affinity capture of glycosylated cell-surface proteins (Wollscheid et al., 2009), to obtain a comprehensive overview of cell-surface markers. In addition, cell-surface protein data sets could be integrated with phosphoproteomic analysis of intracellular proteins (Phanstiel et al., 2011) to generate a detailed understanding of signaling pathways that regulate self-renewal and differentiation.

## Experimental Procedures

### Cell Lines

ES cell lines R1 (129X1 × 129S1; passages 12–16) (Nagy et al., 1993) and E14TG2a (129P2/OlaHsd; passages 18–22) (Hooper et al., 1987), TS cell lines F4 (Institute for Cancer Research [ICR]; passages 12–16) and Rosa (ICR [Gt(ROSA)26Sor / +]; passages 8–12), and XEN cell lines A4 (ICR; passages 10–14) and E4 (ICR; passages 12–15) were derived from E3.5 blastocysts and cultured in the absence of feeder-cells as previously described (Rugg-Gunn et al., 2010). EpiSC lines 129S2 (passages 14–20) and B2 (ICR; passages 8–16) were derived as described (Brons et al., 2007). EpiSC were cultured in N2B27 media (Ying and Smith, 2003) supplemented with 10 ng/ml Activin A and 12 ng/ml bFGF on fibronectin or irradiated mouse embryonic fibroblasts.

*Sox17*-inducible ES cells were generated by electroporation of R1 ES cells with 5 μg of PB-TET-Sox17, 5 μg pCAG-rtTA-Puro, and 100 ng pCAG-PBase (Wang et al., 2008b) followed by puromycin selection (1.25 μg/ml). To induce extraembryonic endoderm differentiation, *Sox17*-ES cells were treated for 12 days with 100 ng/ml doxycycline (Sigma) in ES cell media.

ES cell to EpiSC conversion was accomplished by transferring 50,000 ES cells into one well of a 6-well plate, precoated with fibronectin, in EpiSC media. Media was changed daily and cells passaged after four days. EpiSC to ES cell reprogramming was performed by transferring 500,000 *Nanog*-EpiSC into one well of a six-well plate, precoated with irradiated mouse embryonic fibroblasts, in ES cell media supplemented with 1 μM PD0325901, 3 μM CHIR99021, 1000 U/ml LIF, and 1,000 ng/ml doxycycline (Sigma). *Nanog*-overexpressing EpiSCs (129S2 line) were generated using Lipofectamine 2000 (Invitrogen) and 1 μg of PB-TET-Nanog-ires-GFP, 1 μg pCAG-rtTA-Puro, and 2 μg pCAG-PBase (Wang et al., 2008b) followed by puromycin selection (1.2 μg/ml).

### Mouse Embryos

Embryos were collected at appropriate time points from timed natural matings of ICR outbred mice. Preimplantation embryos were flushed from uteri at E3.5 with M2 media (Millipore). E4.5 embryos were obtained by culturing E3.5 embryos for 24 hr in KSOM supplemented with amino acids (Millipore) at 37°C in 5% CO_2_. E5.5 embryos were dissected from decidua in PBS. All animal work was carried out following Canadian Council on Animal Care Guidelines for Use of Animals in Research and Laboratory Animal Care under protocols approved by the Toronto Centre for Phenogenomics Animal Care Committee.

### Sample Preparation, MudPIT Analyses, and Protein Identification

In situ biotinylation of ES, TS, XEN, and EpiSC was carried out as previously described (Roesli et al., 2006). Sample preparation, digestion, MudPIT analyses and protein identifications were as previously described (Elschenbroich et al., 2009; Taylor et al., 2009). Experimental procedures are detailed in Supplemental Experimental Procedures.

### Flow Cytometry of Cell Lines and Blastocysts

Single-cell suspensions of ES, TS, XEN, and EpiSC were obtained by dissociating with 0.05% trypsin or cell dissociation buffer (Invitrogen) at 37°C. Blastocysts (typically 30–50 per experiment) were treated with acid Tyrode's solution to remove the zona pellucida, then incubated in 2 mg/ml collagenase IV for 20 min at 37°C, followed by Hanks'-based cell dissociation buffer (Invitrogen) for 20 min on ice, then manually dissociated into single cells using a finely pulled glass capillary. Cells were incubated with primary antibody (Supplemental Experimental Procedures) in staining buffer (2% FBS in PBS) for 30 min on ice. Cells were washed once with staining buffer, incubated with secondary antibody in staining buffer for 30 min on ice and washed once in staining buffer. Cells were suspended in 0.2 μg/ml propidium iodide in staining buffer.

For ES, TS, and XEN cell derivation, embryo cells were sorted into ES cell media (Lanner et al., 2010) supplemented with 1 μM PD0325901, 3 μM CHIR99021, and 1000 U/ml LIF (Ying et al., 2008) or TS/XEN cell media (Rugg-Gunn et al., 2010) supplemented with 30 ng/ml FGF4 and 1 μg/ml heparin.

Flow cytometry was performed at the Sickkids - UHN Flow Cytometry Facility using a Becton Dickinson LSR II and Dako Cytomation MoFlo. At least 10,000 final events were recorded for each flow cytometry analysis.

### Gene Expression Analysis

Single cell gene expression analysis was performed using 48.48 Dynamic Arrays on the BioMark System (Fluidigm). Individual cells were flow sorted directly into 5 μl RT-PreAmp Master Mix, containing CellsDirect 2× Reaction Mix (Invitrogen), 0.2× assay pool of recommended TaqMan GeneExpression Assays (20×, Applied Biosystems), and RT/Taq Enzyme (CellsDirect qRT-PCR kit, Invitrogen). Cell lysis, sequence-specific reverse transcription (50°C for 20 min) and sequence-specific amplification (18 cycles of: 95°C for 15 s, 60°C for 4 min) were performed immediately following flow sorting. The preamplified product was diluted 5-fold before being analyzed on 48.48 Dynamic Arrays on the BioMark System with TaqMan GeneExpression Assays (Applied Biosystems). Ct values were calculated using Biomark's Real-time PCR Analysis software. Each reaction for each plate was filtered by using the pass fail quality control metric (Fluidigm) and normalized against *Actb*. Cells with low (*Actb* Ct value > 16) or absent amplification were excluded from the analysis. Reactions that failed due to quality or no product were set to the maximum observed Ct value plus 1. Data were rescaled by subtracting the observed Ct value from the maximum plus 1, which results in a positive scale of increasing value proportional to increasing transcript. Data were analyzed in R. Heat maps were made using the heatmap.2 function from the gplots package, principal component analysis was calculated using the prcomp function and plotted with the plot function.

RNA was extracted from bulk flow-sorted cells using Trizol (Invitrogen) and the RNeasy Micro kit (QIAGEN). RNA (1 μg for cell lines, or entire sample for blastocyst cells) was reverse transcribed using the QuantiTect Reverse Transcription Kit (QIAGEN) and subjected to quantitative PCR analysis as previously described (Rugg-Gunn et al., 2010).

### Statistical Analysis

We used t tests to calculate p values for the fold change difference between the membrane and nonmembrane fractions, based on spectral counts in the replicates for each fraction. p values were corrected for multiple testing using the Benjamini and Hochberg false discovery rate. Enrichment for gene ontology terms was examined using Database for Annotation, Visualization and Integrated Discovery (DAVID) v6.7 software (Dennis et al., 2003). Enrichment value and Fisher's exact p value are indicated in the text. flowClust provides methods for identification of statistically distinct cell populations through modeling of cytometric data (Lo et al., 2009). Optimal models are determined using the Bayesian Information Criterion.

## Figures and Tables

**Table 1:** Functional Classification of the Plasma Membrane Proteins Identified in ES, TS, and XEN Cells

	Unique ES	Unique TS	Unique XEN	Multiple Cell Types
**Signal Transduction**				

Integrin	Itga9	Itga7; Itgb3; Itgb5	−	Itga3; Itga5; Itga6; Itgav; Itgb1; Ptk2; RhoA
FGF	Fgfr1	Fgfr2	Hhip	−
Wnt	Wntlrp1	−	−	Ctnnb1; Slc9a3r1
BMP	Gpc3	Htra1	−	−
TGF-β	−	Htra1	−	Spnb2
Hedgehog	−	−	Hhip	Ctnna1
Notch	−	Notch3	Adam10; Notch2	Ncstn
Insulin	−	Sorbs1	−	Insr
Phosphatase	−	−	−	Ptprb; Ptprf; Ptprg; Ptprk
G-protein	Lgr4; Lphn1; V1rc6	Gnaq	Ric8	Cd97; Gna11; Gna13; Gnai3; Gnas; Gnb2l1; Gnb1; Lphn2; Olfr54; Tacr1
Small GTPase	Pecam1	Rab13	Rheb; Sar1b	Arf1; Arf4; Arf5; Arf6; Arhgap1; Arhgdia; Gna13; Gnb1; Grb2; Rab5a; RhoA; RhoC; Rras2
Second messenger	Ncam1	Gnaq; L1cam	−	Gna11; Gnas; Gnb1; Gnb2l1; Slc9a3r1

**Cell-Cell Adhesion**				

	Enah; Pvrl2; Pvrl3	Arvcf; Cldn3; Dsc2; L1cam; Pkp2	Cdh6; Pdpn	Cadm1; Cdh3; Cdh5; Ctnnb1; Dsg2; Fat1; Icam1; Itga5; Itga6; Lgals1; Mcam; Myh9; Nrcam; Pcdh1; Pnn; Ptk7; Tek; Vasp

**Cell Migration**				

	Alcam; Enah; Epha4; Ephb2; Mmp14; Pecam1	Itgb3; L1cam; Scarb1; Shroom2	B4galt1; Gja1; Pdpn; Robo2	Col18a1; Enpep; Ephb3; Gab1; Gna13; Itga3; Itga6; Lama1; Lama5; Myh10; Nrcam; Pafah1b1; Podxl; Ptk2; Robo1; Rras2; St14; Tek; Vasp; Vcl

The plasma membrane proteins were grouped into signal transduction pathways, cell-cell adhesion, and cell migration functions according to their GO annotation. Given are the gene symbols of the proteins identified. Proteins shown were detected uniquely in one cell type or common to more than one cell type.

**Table 2:** Functional Classification of the Plasma Membrane Proteins Identified in ES Cells and EpiSC

	Unique ES	Unique EpiSC	ES and EpiSC
**Signal Transduction**			

Integrin	Itga9; RhoA	Adam17; Cd47; Itga1; Itgb4; Itgb5; Ptk2	Itga3; Itga5; Itga6; Itgav; Itgb1
FGF	−	−	Fgfr1
Wnt	−	Ror2	Ctnnb1; Lrp1^a^; Slc9a3r1
BMP	−	−	Gpc3
TGF-β	−	−	Spnb2
Hedgehog	−	−	Ctnna1
Notch	−	Adam17; Notch1; Notch2; Notch3	Ncstn; Nle1
Insulin	Insr	−	−
Phosphatase	Ptprb	Ptprd;	Ptprf; Ptprg; Ptprk
G-protein	Lphn1; V1rc6	Gna11; Gna13	Cd97; Gnai3; Gnas; Gnb1; Gnb2l1; Lgr4; Lphn2; Tacr1
Small GTPase	Arf6; Pecam1; RhoA	B230208h17rik; Gna13; Itsn1; Rab12	Arf1; Arf4; Arf5; Arhgap1; Arhgdia; Gnb1; Rab5a; Rab6; Rap1a; RhoC
Second messenger	−	Gna11; L1cam	Gnas; Gnb1; Gnb2l1; Ncam1; Slc9a3r1

**Cell-Cell Adhesion**			

	Pvrl3; Tek	Cdh2; Cdh4; Cdh10; Dsc2; Frem2; L1cam; Pcdh7; Pdpn; Ror2; Shroom3; Vasp	Cadm1; Cdh3; Ctnnb1; Dsg2; Enah; Fat1; Icam1; Itga5; Itga6; Lgals1^a^; Mcam; Myh9; Nrcam; Pcdh1; Pnn; Ptk7; Pvrl2

**Cell Migration**			

	Enpep; Epha4; Ephb2; Gab1; Mmp14; Pecam1; Tek	Cdh2; Cdh4; Erbb2; Gna13; Itga1; Kif5c; L1cam; Nfasc; Pdpn; Ptk2; Shroom2; Vasp	Alcam; Col18a1; Enah; Ephb3; Itga3; Itga6; Lama1^b^; Lama5; Nrcam; Pafah1b1; Podxl; Robo1; Scye1; St14; Vcl

The plasma membrane proteins were grouped into signal transduction pathways, cell-cell adhesion, and cell migration functions, according to their GO annotation. Given are the gene symbols of the proteins identified. Proteins shown were detected uniquely in one cell type or common to more than one cell type.

a EpiSC > ES by 20-fold.

b ES > EpiSC by 20-fold.

## Accession Numbers

Microarray data of ES, EpiSC, TS, and XEN cells have been submitted to Gene Expression Omnibus with the accession number GSE34799. The proteomic data associated with this manuscript may be downloaded from the Proteome Commons (http://www.proteomecommons.org) Tranche network using the following hash code: Er2VCY1Hc+WTIpmr4eqbkjPkSG4y26NVZK5oeAYAPGQbbO3Foi4Ha6eoOUPDdqvAXhjpl7a2J4J63YqtAZ+N1O4yuPEAAAAAAAB/BQ==.

## Supplemental Information

Document S1. Figures S1–S3 and Supplemental Experimental Procedures

Table S1. Complete Protein Data Set for ES, TS, XEN and EpiSC, Related to Figure 1A

Table S2. Final Cell-Surface Protein Data Set for ES, TS, XEN and EpiSC, Related to Figures 2A and 4A

Table S3. Gene Ontology Terms for Final Cell-Surface Protein Data Set and Membrane-Depleted Whole-Cell Fraction, Related to Figure 1C

Table S4. Protein and RNA Expression Data for All Proteins Detected in ES, TS, XEN, and EpiSC, Related to Figure 1D
