# Supplementary material for: Cell-Surface Proteomics Identifies Lineage-Specific Markers of Embryo-Derived Stem Cells
Source: Dev Cell. 2012 Apr 17;22(4):887–901. doi: 10.1016/j.devcel.2012.01.005 (PMC3405530; doi:10.1016/j.devcel.2012.01.005)
Supplement: Document S1. Figures S1–S3 and Supplemental Experimental Procedures [file mmc1.pdf]

Developmental Cell, Volume *22*

Supplemental Information

Cell-Surface Proteomics Identifies Lineage-Specific Markers of Embryo-Derived Stem Cells

Peter J. Rugg-Gunn, Brian J. Cox, Fredrik Lanner, Parveen Sharma, Vladimir Ignatchenko, Angela C.H. McDonald, Jodi Garner, Anthony O. Gramolini, Janet Rossant, and Thomas Kislinger

Figure S1, related to Figures 2B, 2C and 2D	p. 2
Figure S2, related to Figure 2E	p. 3
Figure S3, related to Figure 5	p. 4
Table S1, related to Figure 1A	p. 5 (separate file)
Table S2, related to Figures 2A and 3A	p. 5 (separate file)
Table S3, related to Figure 1C	p. 5 (separate file)
Table S4, related to Figure 1D	p. 6 (separate file)
Supplemental experimental procedures	p. 7-15
Supplemental references	p. 16

Figure S1

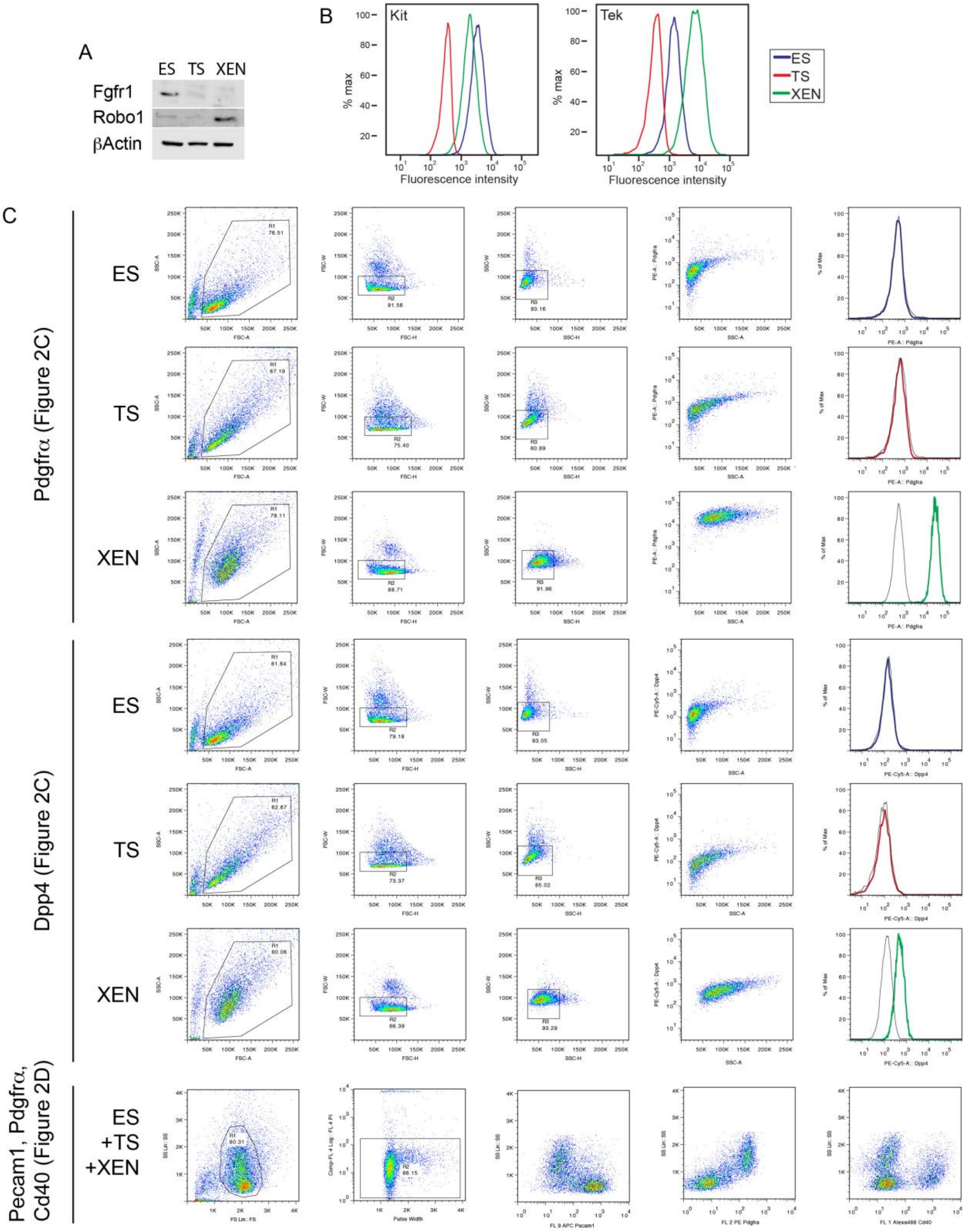

Figure S1, related to Figures 2B, 2C and 2D. Analysis of cell-surface proteins in stem cell lines. (A) Western blot demonstrating cell-specific expression of Fgfr1 (ES cells) and Robo1 (XEN cells). These antibodies failed to give a signal by immunoflureorescence or by flow cytometry. (B) Flow cytometry validation of two proteins, Kit and Tek, which were detected by mass spectrometry in two cell lines (ES and XEN cells). These protein markers may be useful for negative TS cell selection. (C) Gates used for Figures 2C and 2D. Unstained controls are shown as black line in histograms.

Figure S2

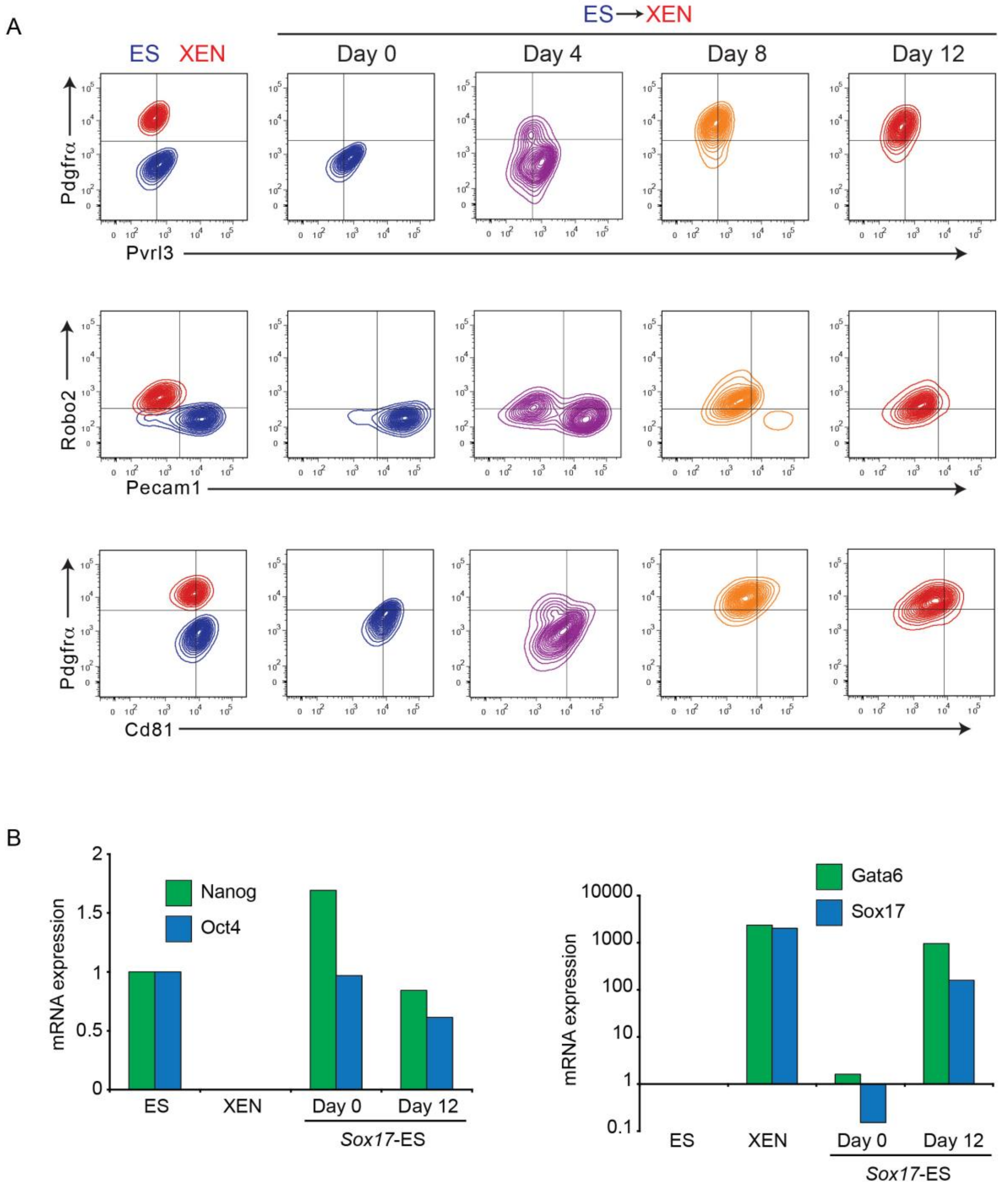

Figure S2, related to Figure 2E. Identified cell-surface proteins can be used to investigate cell fate changes during differentiation. ES cell to XEN cell conversion was induced by forcing *Sox17* expression in ES cells for 12 days. (A) Cell-surface markers of ES (Pecam1, Pvrl3 and Cd81) and XEN (Robo2 and Pdgfrα) were monitored by flow cytometry every four days. (B) qRT-PCR revealed that *Sox17* induction resulted in downregulation of ES cell factors *Nanog* and *Oct4*, and upegulation of XEN cell factors *Gata6* and *Sox17*. Similar results were obtained with a second *Sox17*-ES cell line (not shown).

Figure S3

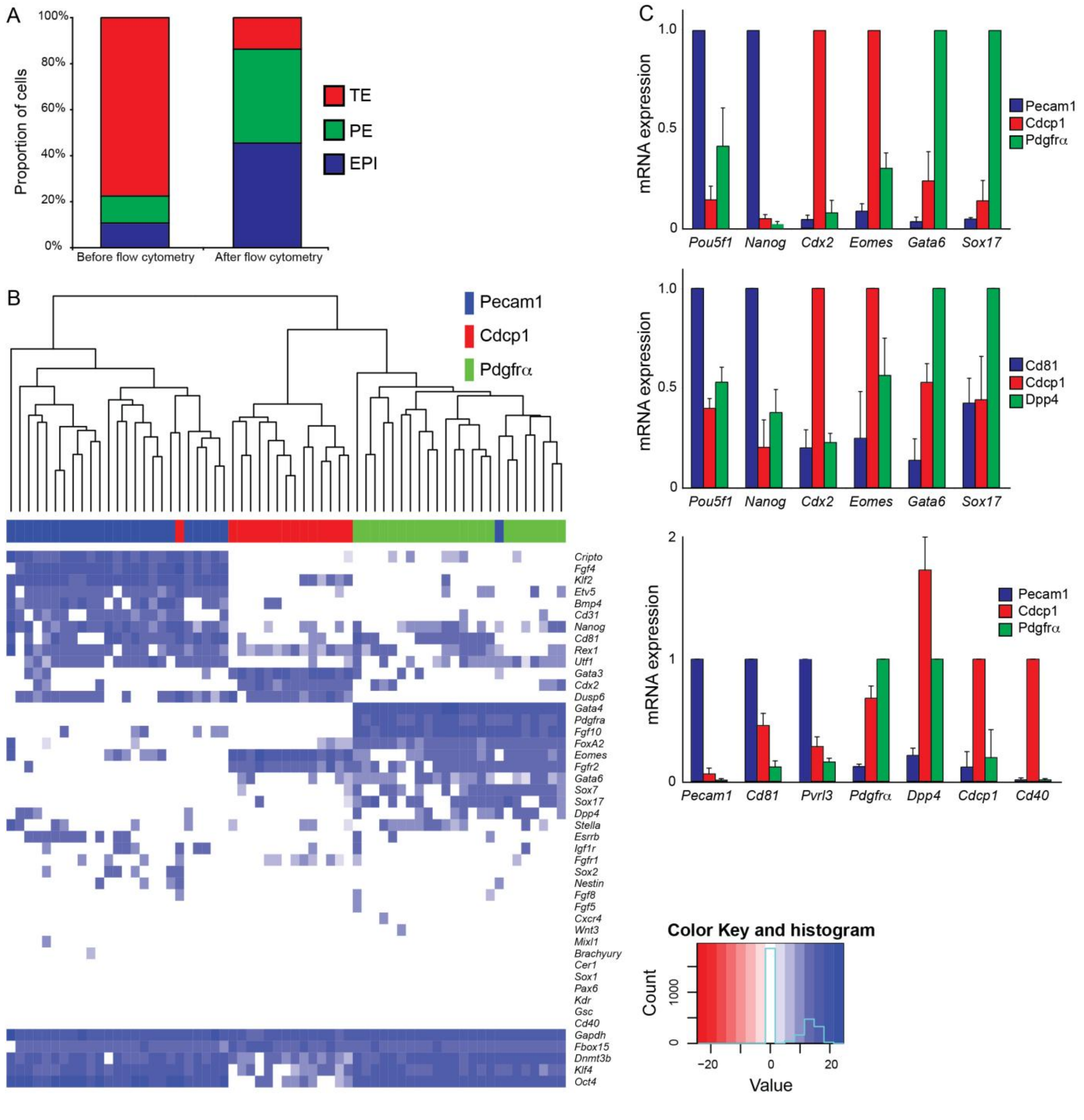

Figure S3, related to Figure 5. Gene expression profiles of EPI, TE and PE. (A) Loss of TE after embryo dissociation and flow cytometry. The proportion of EPI, PE and TE in unpertubed E4.5 embryos was measured using immunofluorescence for lineage-specific factors Nanog, Gata6 and Cdx2. After flow cytomtery, the proportion of TE is reduced compared to EPI and PE. (B) Heat map of expression levels for 48 genes from 63 cells. Individual cells were flow sorted from E4.5 embryos into EPI (Pecam1 positive), PE (Pdgfrα positive) or TE (Cdcp1 positive) and analyzed using the BioMark System. Cells clustered into EPI, PE and TE according to their gene expression profiles. (C) Cells isolated from E4.5 embryos were sorted by flow cytometry using a combination of antibodies against either Pecam1/Cdcp1/Pdgfrα or Cd81/Cdcp1/Dpp4 and analyzed by qRT-PCR for gene expression levels of known lineage-specific transcription factors and cell-surface proteins. Gene expression levels were normalized to the sample with highest expression. Bars, s.d. (n=2 biological replicates).

Table S1, related to Figure 1A. Complete protein data set for ES, TS, XEN and EpiSC.

(see accompanying Excel file)

Raw spectral counts for membrane-enriched (e.g. ES.1) and whole-cell fractions (e.g. ESH.1) are shown for all 32 MudPIT runs. Normalized mean values are indicated. As the replicates are paired no permutations of these ratios were made.

Table S2, related to related to Figures 2A and 4A. Final cell-surface protein data set for ES, TS, XEN and EpiSC.

(see accompanying Excel file)

Normalized spectral counts for membrane-enriched and whole-cell fractions are shown. Data are presented for all four cell lines together and also individually.

Table S3, related to Figure 1C. Gene ontology terms for final cell-surface protein data set and membrane-depleted whole-cell fraction.

(see accompanying Excel file)

Proteins predicted to be localized to the cell-surface were strongly enriched for functional classes that are characteristic of plasma membrane proteins, including transmembrane receptor tyrosine kinases, cell adhesion and cell migration. Gene ontology categories and terms are shown, along with the number (Count) and IPI designator (Genes) of genes within each term. Fold enrichment, Fisher exact P-value, and Bonferroni, Benjamini and FDR corrected P-values were calculated using the DAVID Bioinformatics Resource (http://david.abcc.ncifcrf.gov/).

Table S4, related to Figure 1D. Protein and RNA expression data for all proteins detected in ES, TS, XEN and EpiSC.

(see accompanying Excel file)

Normalized spectral counts for membrane-enriched and whole-cell fractions are shown, together with microarray values for all proteins detected. Cell-surface prediction column designates those proteins that are predicted to be at the plasma membrane.

Supplemental Experimental Procedures

Biotinylation of cell-surface proteins

In situ biotinylation of ES, TS, XEN and EpiSC was carried out as previously described (Roesli et al., 2006). In brief, plated cells were washed three times with ice cold phosphate buffered saline (PBS). Cells were then incubated with 150 µM EZ-link sulfo-NHS-SS-Biotin (Pierce Biotechnology) for 5 min at room temperature. The biotinylation reaction was then terminated by the addition of 150 mM Tris-HCl. Cells were harvested by scraping the cells into PBS containing 150 µM oxidised glutathione and collected by centrifugation at 1,000g. The cell pellet was washed once in PBS containing 150 µM oxidised glutathione, then lysed for 30 min on ice in lysis buffer (2% NP-40, 2% SDS, 100 µM oxidised glutathione and protease inhibitors; Complete, EDTA-free, Roche Diagnostics). Cell lysates were then cleared by centrifugation at 16,100g.

Protein Precipitation and Digestion

Biotinylated proteins were captured on streptavidin sepharose beads (Pierce Biotechnology). A slurry of 600 µl of beads was washed three times in buffer A (1% NP-40, 0.1% SDS and 20 mM oxidised glutathione in PBS) and added to the cleared lysate and allowed to rotate for 2 hours at 4°C. Unbound proteins were removed by washing three times with buffer A, two times with buffer B (2 M NaCl, 0.1% SDS and 20 mM oxidised glutathione in PBS) and two times with 50 mM Tris-HCl. Proteins were eluted three times with 400 µl of 5% β-mercaptoethanol in PBS for 30 min each at 37°C. 100 µg of protein per sample, as determined by Bio-Rad protein assay (Bio-Laboratories), were precipitated in 10% TCA in acetone at -20°C overnight. Proteins were pelleted by centrifugation at 10,000g for 30 min, washed in 100% acetone and centrifuged at 10,000g for 30 min. The supernatant was decanted and remaining acetone evaporated at 37°C. The dried protein pellet was resuspended in 50 µl freshly

prepared re-suspension buffer (8 M urea in 100 mM Tris pH8.5 containing 2 mM DTT) and placed in an incubator at 37°C for 30 min. Free thiol moieties were alkylated by adding iodoacetamide (final concentration 8 mM) for 2 h at 37°C. The sample was then diluted to a urea concentration of ~1.5 M with 100 mM ammonium bicarbonate buffer (pH8.5) and calcium chloride was added to a final concentration of 2 mM. Proteins were digested with a 1:40 ratio of proteomics grade trypsin (Promega) at 37°C overnight. Protein digestion was stopped by addition of 50 µL 2.5% TFA. For peptide concentration and removal of salts and detergents, samples were solid phase extracted using C18 MacroSpin Columns (The Nest Group) according to the manufacturer's instructions. Eluted peptide mixtures were vacuum dried and reconstituted with 40 µl 5% acetonitrile 0.1% formic acid.

Mass Spectrometry

A fully automated 9-cycle MudPIT was performed as described (Elschenbroich et al., 2009; Taylor et al., 2009). Briefly, a nano-HPLC (Proxeon Biosystems) was interfaced with an LTQ-Orbitrap XL mass spectrometer (Thermo Fisher Scientific), which is equipped with a nano-electrospray source (Proxeon Biosystems). An analytical column was made by pulling a 75 µm i.d. fused silica microcapillary (Innova-Quartz) column using a P2000 laser puller (Sutter Instruments). The column was then packed with ~7 cm of 5 µ Magic C18 100Ǻ reversed phase material (Michrom Bioresources Inc.) with an in-house pressure vessel. A Kasil fritted pre-column (150 µm i.d.) was packed with ~4 cm of 5 µ Magic C18 100Ǻ reversed phase material (Michrom Bioresources Inc) followed by ~ 4 cm of Luna® 5µ SCX 100Ǻ strong cation exchange resin (Phenomenex) by means of an in-house pressure vessel. This vented-column set-up was placed in-line with the EasyLC system and connected via a microsplitter tee (Proxeon Biosystems) to which a distal voltage of 2.2 kV was applied. Acidified sample was then automatically loaded from a 96-well microplate autosampler using the EASY-nLC system. The sample loading was followed by a 120 min HPLC gradient consisting of buffer A (water/0.1% formic acid) and buffer B (acetonitrile/0.1% formic acid) at a flow of 400 nl/min, similar as recently reported

(Elschenbroich et al., 2009; Taylor et al., 2009). After the sample loading step, consecutive loading ("salt bumps") of 8 µl of 25 mM, 50 mM, 75 mM, 100 mM, 125 mM, 150 mM, 200 mM, and 500 mM NH_4Ac was followed by the same HPLC gradient as above.

We performed a total of 32 MudPIT runs: four runs for the membrane-enriched fraction and four runs for the membrane-depleted whole-cell fraction for each cell type.

The MS functions were controlled by the XCalibur data system (Thermo Fisher Scientific) and the chromatographic conditions by the Easy-LC software. All samples were analyzed on a LTQ-Orbitrap XL. The instrument method consisted of one MS full scan (400-1800 m/z) in the Orbitrap mass analyzer, an automatic gain control target (AGC target) of 500,000 with a maximum ion injection of 500 ms, 1 microscan and a resolution of 60,000 and using the preview scan option. Five data-dependent MS/MS scans were performed in the linear ion trap using the five most intense ions at 35% normalized collision energy. AGC targets for the LTQ were 10,000 with a maximum ion injection time of 100 ms. A minimum ion intensity of 1,000 was required to trigger a MS/MS spectrum. The dynamic exclusion was applied using a maximum exclusion list of 500 with one repeat count with a repeat duration of 30 seconds and exclusion duration of 45 seconds.

Protein identification

Raw data was converted to m/z XML using ReAdW and searched by X!Tandem against a locally installed version of a mouse IPI (http://www.ebi.ac.uk/IPI) protein sequence database (version 3.41, released March 18, 2009). The search was performed with a fragment ion mass tolerance of 0.4 Da, a parent ion mass tolerance of ±10 ppm. Complete tryptic digest was assumed. Carbamidomethylation of cysteine was specified as fixed, and oxidation of methionine as variable modification. To estimate and minimize our false positive rate the protein sequence database also contained every IPI protein

sequence in its reversed amino acid orientation (target-decoy strategy) as recently described (Cox et al., 2009). In the current study we applied cut-off criteria resulting in 1.5-2.5% decoy protein identification per analysed sample type.

Proteomics data analysis

All data transformations and calculations were done using R scripts. Filtered proteomics data were stored as MySQL databases and merge with annotation information from other public databases. The spectral counts for all proteins in the data set were normalized by scaling to the global mean spectral counts. Replicates were then summed to generate a final table of spectral counts, used for quantitative comparisons. To calculate the fold change and associated false positive discovery corrected P-value the mean fold change of the four replicate membrane samples versus the four replicate membrane depleted whole cell extracts was calculated. The mean fold change and the distribution of the fold change values were then used to calculate a P-value, which was then corrected using the Benjami-Hershberg false positive discovery rate correction (FDR).

Machine learning modeling of plasma membrane proteins

Prediction of transmembrane (TM) helices and signal peptides using protein annotation data has been used previously to segregate plasma membrane proteins from non-plasma membrane proteins (Nunomura et al., 2005). We applied this TM prediction method to our data set and assessed its performance using a subset (n = 288) of proteins with known locations retrieved from UniProt. This revealed that 49% of true plasma membrane proteins were selected as false positives due to the absence of a predicted TM helix. In particular, this resulted in the exclusion of many single pass TM proteins and GPI-anchored proteins. For our data, this approach generated a sensitivity of only 0.5 and a specificity of 0.9, and re-analysis of the Nunomura et al. (2005) data gave similar values (sensitivity = 0.6, specificity = 0.7). Thus, analysis of protein data sets using TM prediction models may introduce

bias towards multi-pass TM proteins at the expense of other plasma membrane localized proteins, resulting in low protein sensitivity.

To improve this analysis, we developed a novel integrated data mining strategy, which generated high-quality data with substantially improved sensitivity (0.85) and similar specificity (0.7) to the TM prediction analysis. Membrane-depleted whole-cell lysate data were generated and a decision stump machine learning model was applied to select a fold ratio of spectral counts of membrane-enriched / membrane-depleted whole-cell fractions. All machine learning analysis was done with the WEKA package. Cell-specific tables of mean fold change and $\log_2$ transformed spectral counts were generated. Gene ontology (GO) cellular component terms and molecular function terms were used to assign a cellular location (class) to each protein. The class was assigned only if it could be uniquely assigned to a single cellular component, otherwise it was categorized as mixed class. If no suitable annotation was found then the protein was categorized as unknown. Only GO annotation that had evidence codes for "inferred from direct assay" were taken. All proteins with a class other than plasma membrane were assigned to the class "other". The training set was then split off from this table as all proteins with a spectral counts value greater than zero in either cell fraction (membrane-enriched or whole cell) and belonging to the class of other or plasma membrane. The remainders were the set of proteins to be predicted. Given the simple nature of the data set the machine learning method description stump was used with Bagging. A ten-fold cross validation procedure and a 66% split of the training set were both used to test the models. Class prediction values for each protein and cell line were then stored as a MySQL database and queried against a database of expression values with associated fold change and FDR corrected P-values to determine those proteins that were predicted to be plasma membrane and were statistically above the fold change cut off given by the model.

Immunofluorescence

ES, TS, XEN and EpiSC were grown on glass chamber culture slides (BD-Falcon). For visualisation of the biotin epitope, biotinylated cells were washed in PBS and fixed in 2% paraformaldehyde (PBS pH7.0). Cells were blocked with 5% FBS in permeabilization buffer (0.2% Tween-20, 0.5% Triton X-100 in PBS pH7.0) for 30 min and incubated with anti-biotin antibody (Jackson ImmunoResearch) in permeabilization buffer. Cells were washed three times with PBS and incubated with AlexaFluor 633-conjugated secondary antibody (Invitrogen). Images were collected by using a Leica DM IRBE inverted microscope equipped with a Leica TCS SP laser scanning confocal system. For visualisation of cell-surface proteins, cells were washed once in PBS and fixed in 2% paraformaldehyde (PBS pH7.0). Cells were blocked with 10% FBS in PBS for 1 hour and incubated with primary antibody in blocking buffer overnight at 4°C. Cells were washed three times with PBS and incubated with secondary antibody for two hours at room temperature. Nuclei were stained with Hoechst 33342 (1 μg/ml, Invitrogen). Images were collected using a Zeiss Aviovert 200M. Embryos were fixed in 2% paraformaldehyde (PBS pH 7.0) for two hours at 4°C, then permeabilised in 0.5% Triton X-100 in PBS for 15 min. Embryos were blocked in 5% FBS in PBS for one hour at room temperature and incubated with primary antibody overnight at 4°C. Embryos were washed three times with PBS and incubated with secondary antibody for two hours at room temperature. Nuclei were stained with Hoechst 33342 (1 μg/ml, Invitrogen). Images were collected on a Quorum spinning disc confocal microscope equipped with a Hamamatsu C9100-13 EM-CCD. The objective lens used was 25X water, NA=0.80. Optical section thickness ranged from 1-5 μm. Velocity software (Perkin-Elmer) was used to apply deconvolution algorithms to remove out of focus signal. ImageJ software was used to generate stacked images.

DNA methylation analysis of *Stella* promoter region

Genomic DNA from ~10,000 flow-sorted cells was bisulfite-modified using the EpiTect Bisulfite Kit (Qiagen) following the manufacturer's instructions. Modified DNA (25% of total eluate) was amplified with primers specific to bisulfite-converted DNA using HotStartTaq (Qiagen) for 40 cycles with an annealing temperature of 53ºC. Primer sequences are TTTGTGATTAGGGTTGGTTTAGAAT and ACCAAAACCCAATTAACAATCAAT. The region analyzed spans six CpG dinucleotides in the *Stella* promoter region corresponding to -267bp to -137bp from the transcriptional start site. PCR products were cloned into TOPO pCRII (Invitrogen) and sequenced. At least ten unique clones were analyzed for each cell type.

Primary antibodies:

Antibody	Company	Catalogue no.	Species	Dilution for IF	Dilution for FACS
Ephb4	R&D Systems	AF446	Goat	1 in 100	1 in 50
Gpc3	Santa Cruz	sc17613	Goat	1 in 500	1 in 50
Pvrl3	Abcam	ab16913	Rat	1 in 100	1 in 50
Pecam1	BD Biosciences	553370	Rat	1 in 400	-
Pecam1-APC	BD Biosciences	551262	Rat	-	1 in 50
Pecam1-Biotin	BD Biosciences	553371	Rat	1 in 400	-
Pecam1-PE/Cy7	BioLegend	102418	Rat	-	1 in 50
Cd47-APC	Abcam	ab95603	Rat	1 in 100	1 in 50
Cd81	R&D Systems	MAB4865	Rat	1 in 200	1 in 200
Cd81-FITC	R&D Systems	FAB4865F	Rat	-	1 in 20
Cd40	R&D Systems	AF440	Goat	1 in 500	1 in 50
Fgfr2	Santa Cruz	sc122	Rabbit	1 in 1000	1 in 200
Cd117-APC	BD Biosciences	651074	Rat	1 in 100	1 in 50
Tek-PE	E-Bioscience	12-5987	Rat	1 in 100	1 in 100
Cdh10	Gift from Dr. Peter Clark, Imperial College		Rabbit	1 in 50	-
Mmp14	Abcam	ab51074	Rabbit	1 in 100	1 in 50
Sirpa-Biotin	Abcam	ab95644	Rat	1 in 100	1 in 200
Fgfr1	Santa Cruz	sc121	Rabbit	-	-
Notch3-PE	Abcam	ab95775	Hamster	1 in 50	1 in 50
Cdh2	Abcam	ab98952	Mouse	1 in 100	1 in 100
Cdcp1	R&D Systems	AF4515	Sheep	1 in 400	1 in 100
Mertk	R&D Systems	AF591	Goat	1 in 200	1 in 50
Robo1	Abcam	ab7279	Rabbit	-	-
Robo2	Santa Cruz	sc16615	Goat	1 in 100	1 in 50
Plxna4	Abcam	ab39350	Rabbit	1 in 100	1 in 100
Cdh6	Santa Cruz	sc1503	Goat	1 in 500	1 in 50
Ggt1	Abcam	ab55138	Mouse	1 in 200	1 in 200
Scarb1	Abcam	ab396	Rabbit	1 in 500	1 in 200
Pdgfra	E-Bioscience	14-1401	Rat	1 in 100	-
Pdgfra-PE	E-Bioscience	12-1401	Rat	-	1 in 200
Dpp4	AbD Serotec	MCA2345	Rat	1 in 100	1 in 200
Dpp4-RPE	AbD Serotec	MCA2345PE	Rat	-	1 in 10
Cd44-APC	E-Bioscience	17-0441-81	Rat	1 in 50	1 in 250
Cdx2	BioGenex	MU392-UC	Mouse	1 in 200	-
Gata6	R&D Systems	AF1700	Goat	1 in 400	-
Nanog	Reprocell	RCAB0002	Rabbit	1 in 200	-
Klf4	R&D Systems	AF3158	Goat	1 in 1200	-
Oct4	Santa Cruz	sc5279	Mouse	1 in 100	-

The following antibodies failed due to absence of signal or multiple bands on a Western blot:

Antibody	Company	Catalogue no.
Epha4	R&D Systems	AF641
Ephb2	R&D Systems	AF467
Itga9	R&D Systems	AF3827
Spn	Abcam	ab21853
Erbb2	Abcam	ab16901
Frem2	Abcam	ab75803
Gpc2	R&D Systems	AF2355
Ocln	Abcam	ab31721
Plxnb1	Abcam	ab39717
Dsc2	Abcam	ab76628
Itgb3	Abcam	ab47584
Vasn	Santa Cruz	sc47564
Anpep	Abcam	ab33480
Enpp3	Abcam	ab55580
Ncam1	Sigma	C9672
Sdc1	BD Biosciences	347193
Mcam	Abcam	ab75769
Egfr	Santa Cruz	sc03
Plxnb2	Abcam	ab95804
Enpep	Abcam	ab25009
Ephb3	R&D Systems	AF432
L1cam	Abcam	ab24345
Prdx6	Abcam	ab16824
Vasp	Abcam	ab26650
Flrt2	R&D Systems	AF2877

Secondary antibodies:

Antibody	Dilution for IF	Dilution for FACS
Donkey anti-Goat Alexa Fluor 488 (Invitrogen)	1 in 400	1 in 1000
Donkey anti-Rat Cy3 (Jackson)	1 in 400	1 in 1000
Donkey anti-Rat Cy5 (Jackson)	1 in 400	1 in 1000
Donkey anti-Rabbit Dylight 488 (Jackson)	1 in 400	1 in 1000
Donkey anti-Rabbit Dylight 549 (Jackson)	1 in 400	1 in 1000
Donkey anti-Rabbit Alexa Fluor 647 (Invitrogen)	1 in 400	1 in 1000
Donkey anti-Sheep Alexa Fluor 488 (Invitrogen)	1 in 400	1 in 1000
Donkey anti-Sheep Alexa Fluor 647 (Invitrogen)	1 in 400	1 in 1000
Donkey anti-Mouse Dylight 488 (Jackson)	1 in 400	1 in 1000
Donkey anti-Mouse Dylight 549 (Jackson)	1 in 400	1 in 1000
Streptavidin Alexa Fluor 647 (Invitrogen)	1 in 400	1 in 1000

Supplemental References

Cox, B., Kotlyar, M., Evangelou, A.I., Ignatchenko, V., Ignatchenko, A., Whiteley, K., Jurisica, I., Adamson, S.L., Rossant, J., and Kislinger, T. (2009). Comparative systems biology of human and mouse as a tool to guide the modeling of human placental pathology. Mol. Sys. Biol. *5*, 279.

Elschenbroich, S., Ignatchenko, V., Sharma, P., Schmitt-Ulms, G., Gramolini, A.O., and Kislinger, T. (2009). Peptide separations by on-line MudPIT compared to isoelectric focusing in an off-gel format: application to a membrane-enriched fraction from C2C12 mouse skeletal muscle cells. J. Prot. Res. *8*, 4860-4869.

Nunomura, K., Nagano, K., Itagaki, C., Taoka, M., Okamura, N., Yamauchi, Y., Sugano, S., Takahashi, N., Izumi, T., and Isobe, T. (2005). Cell surface labeling and mass spectrometry reveal diversity of cell surface markers and signaling molecules expressed in undifferentiated mouse embryonic stem cells. Mol. Cell. Prot. *4*, 1968-1976.

Roesli, C., Neri, D., and Rybak, J.N. (2006). In vivo protein biotinylation and sample preparation for the proteomic identification of organ- and disease-specific antigens accessible from the vasculature. Nat. Prot. *1*, 192-199.

Rugg-Gunn, P.J., Cox, B.J., Ralston, A., and Rossant, J. (2010). Distinct histone modifications in stem cell lines and tissue lineages from the early mouse embryo. Proc. Natl. Acad. Sci. USA *107*, 10783-10790.

Taylor, P., Nielsen, P.A., Trelle, M.B., Horning, O.B., Andersen, M.B., Vorm, O., Moran, M.F., and Kislinger, T. (2009). Automated 2D peptide separation on a 1D nano-LC-MS system. J. Prot. Res. *8*, 1610-1616.
